# Differential Effects of *n*-3 and *n*-6 Polyunsaturated Fatty Acids on Placental and Embryonic Growth and Development in Diabetic Pregnant Mice

**DOI:** 10.3390/nu16081182

**Published:** 2024-04-16

**Authors:** Huiying Li, Chuanjing Chen, Shiyi Liu, Yan Shi, Xiaotong Kuang, Xiaolei Song, Duo Li, Kelei Li

**Affiliations:** 1 Institute of Nutrition and Health, Qingdao University, 308 Ningxia Road, Qingdao 266071, China; lihuiying97@126.com (H.L.); 13096291607@163.com (S.L.); jnzqzyysy@126.com (Y.S.); 2017021621@qdu.edu.cn (X.K.); s2826206828@163.com (X.S.); duoli@qdu.edu.cn (D.L.); 2 School of Public Health, Qingdao University, 308 Ningxia Road, Qingdao 266071, China; chuanjingchen@163.com

**Keywords:** maternal diabetes, *n*-3 polyunsaturated fatty acid, embryonic growth, placental growth, fetal growth restriction, metabolomics

## Abstract

The present study aimed to investigate the differential effects of *n*-3 and *n*-6 polyunsaturated fatty acids (PUFAs) on placental and embryonic development. Pregnant mice were assigned to five groups: healthy control (HC), diabetes mellitus control (DMC), diabetes + low-dose *n*-3 PUFA (L*n*-3), diabetes + high-dose *n*-3 PUFA (H*n*-3), and diabetes + *n*-6 PUFA (*n*-6). On E12.5d, the H*n*-3 group, but not the *n*-6 group, had a higher placenta weight. The weight ratio of embryo to placenta in the *n*-6 group was significantly lower than in the H*n*-3 group but higher than in the DMC group. The H*n*-3 group had significantly higher protein levels of VEGF, IGF-1, and IGFBP3, while the *n*-6 group had lower VEGF than the DMC group. Compared with the DMC group, embryonic Cer-16:0 was significantly higher in the H*n*-3 group, while embryonic PC (36:6), PC (38:7), and PE (40:7) were significantly lower in the *n*-6 group. The embryo and placenta weights were positively correlated with placental VEGF, IGFBP3, and embryonic Cer-16:0, and they were negatively correlated with embryonic PC (36:6) and PE (40:7). The weight ratio of embryo to placenta was negatively correlated with embryonic PC (36:6). In addition, embryonic Cer-16:0 was positively correlated with placental VEGF and IGFBP3. In conclusion, *n*-3 PUFA and *n*-6 PUFA improved placental and embryonic growth through different mechanisms.

## 1. Introduction

Maternal diabetes influences about 14% of all pregnancies [1]. It is associated with an increased risk of gestational hypertension, pre-eclampsia, macrosomia, and preterm delivery [2]. In recent years, the adverse effect of maternal diabetes on offspring growth in early life (1000 days comprising gestation and the first two years of postnatal life) has been paid much attention. Our previous animal study observed that STZ-induced maternal diabetes caused fetal growth restriction (FGR) [3]. FGR can result in higher intrauterine and perinatal mortality and is associated with an increased risk of obesity, cardiovascular disease, and chronic kidney disease in later life [4]. Therefore, it is crucial to effectively prevent FGR. 

Fatty acids and lipids are necessary for embryonic growth and development. They can be used as an energy source by oxidation in both the embryo and placenta [5,6,7]. Polyunsaturated fatty acids (PUFAs), especially C20:4*n*-6 and C22:6*n*-3, are important structural components of the nervous system [8,9]. In humans, a lower fetal/maternal ratio of C20:4*n*-6 and C22:6*n*-3 was observed in cases of FGR than normal controls [10], and long-chain (LC) PUFA intake can promote fetal growth [11,12]. Our previous mouse study observed that the supplementation of LC *n*-3 PUFAs (mainly C20:5*n*-3 and C22:6*n*-3) or C18:2*n*-6 (the precursor of C20:4*n*-6) can effectively improve FGR in STZ-induced diabetic pregnancy [3]. However, the mechanism is still unclear. Metabolomic and lipidomic studies in maternal or cord blood and placenta showed a disturbed lipid metabolism in FGR, such as elevated phosphocholine, myoinositol, and sphingosine in the placenta, lower IDL and HDL in maternal blood, and higher LDL, IDL, and VLDL in cord blood [13,14,15]. However, little is known about the influence of PUFA supplementation on the metabolomic profile of growth-restricted embryos and its relationship with embryonic growth.

Free fatty acids can be transferred from mother to fetus across the placenta via free diffusion (such as short- and medium-chain fatty acids) or by facilitated transport (such as LC fatty acids) [16,17], and esterified fatty acids need to be hydrolyzed by placental lipoprotein lipase before transfer. Importantly, the placenta can preferentially transfer C20:4*n*-6 and C22:6*n*-3 from the mother to the fetus to satisfy the requirement of fetal growth through several placental proteins, such as the plasma membrane fatty acid-binding protein (p-FABPpm) and fatty acid transport protein (FATP) [7,17]. Therefore, the normal functioning of the placenta is necessary for the mother-to-fetus transfer of fatty acids. On the other hand, fatty acids may also influence embryonic growth by modulating placental development and function. Several growth factors are involved in the normal angiogenesis and function of the placenta, such as the vascular endothelial growth factor (VEGF), placental growth factor (PGF), insulin-like growth factor-I (IGF-1), and IGF-binding protein 3 (IGFBP3) [18,19], and lower levels of these factors have been associated with FGR in humans [20,21,22,23]. Previous studies showed that *n*-3 PUFA increased serum IGF-1 levels in mice and skeletal muscle IGF-1 mRNA levels in pigs [24,25]. An in vitro study observed that C22:6*n*-3, but not C20:4*n*-6, stimulated VEGF mRNA expression and protein secretion in the first-trimester trophoblast cells [26], while another study found that *n*-6 PUFA increased the VEGF concentration in the skin wounds of rats [27]. The evidence above indicates that PUFA may promote fetal growth by upregulating the expression of these growth factors in the placenta.

In the present study, we aimed to investigate the differential effect of *n*-3 and *n*-6 PUFA supplementation during pregnancy on placental and embryonic development and to explore the potential mechanism by evaluating their effects on placental growth factors and embryonic metabolites.

## 2. Materials and Methods

### 2.1. Study Design

The animals studied were from a previous study evaluating the effect of PUFAs on the incidence of neural tube defects in pregnant diabetic mice, and a detailed experimental design was described in our previous study [3]. Briefly, eight-week-old ICR clean mice were housed in standard laboratory cages in a specific pathogen-free room under standard conditions (21–23 °C, 50–60% humidity, and 12 h light/dark cycle). After one-week adaption, the females were mated with males (3/1), and the pregnant mice were randomized into 5 groups: healthy mice + normal diet (AIN-93G) group (HC, *n* = 6), diabetic mice + normal diet group (DMC, *n* = 9), diabetes + diet of low-dose *n*-3 PUFA group (L*n*-3, *n* = 7), diabetes + diet of high-dose *n*-3 PUFA group (H*n*-3, *n* = 9), and diabetes + diet of *n*-6 PUFA group (*n*-6, *n* = 11). On E6.5d, the mice in the DMC, L*n*-3, H*n*-3, and *n*-6 groups were intraperitoneally injected with 200 mg kg^−1^ STZ (dissolved in 0.1 M sodium citrate buffer, pH = 4.5) to induce diabetes (FBG at E8.5d > 11.1 mmol L^−1^). On E12.5d, the pregnant mice were sacrificed; the embryos were used for metabolomic analysis, and the placentas were used for the analysis of protein levels. The embryo and placenta weights were used to evaluate their intrauterine growth. The weight ratio of embryo to placenta was calculated, which indicates the efficiency of the placenta in supporting embryonic growth, as described previously, and helps estimate the potential risks for chronic diseases in later life [28].

We used the AIN-93G diet as the normal diet, containing 20% casein, 39.75% corn starch, 13.2% maltodextrin, 10% sucrose, 0.3% L-Cystine, 5% cellulose, 7% soybean oil, 3.5% mineral mix S10022G, 1% vitamin mix V10037, and 0.25% choline tartrate (*w*/*w*). The *n*-3 or *n*-6 PUFAs were given to the mice through the diet. The low-dose and high-dose *n*-3 PUFA diets were prepared by replacing 25% soybean oil and 50% soybean oil in the AIN-93G diet with fish oil, respectively. The *n*-6 PUFA diet was prepared by replacing all soybean oil in the AIN-93G diet with corn oil. The fatty acid composition of these 4 diets was described in our published paper [3]. Briefly, the high-dose *n*-3 PUFA diet contained 44.35% total *n*-3 PUFA (% in total fatty acids), including 36.25% C22:6n-3, 5.36% C20:5n-3, and 2.74% C18:3n-3; the *n*-6 PUFA diet contained 54.31% C18:2n-6 and 0.87% C18:3n-3; the normal diet contained 49.78% C18:2n-6 and 6.1% C18:3n-3 [3]. The average amount of daily chow intake was comparable among the different groups [3]. 

### 2.2. Western Blotting Analysis

Proteins were separated using SDS-PAGE and transferred to PVDF membranes (IPVH00010, Millipore, Billerica, MA, USA). The membranes were incubated overnight at 4 °C with primary antibodies, as follows: IGF-1 (1:500, A0830, ABclonal, Woburn, MA, USA), IGFBP3 (1:2000, ab220429, Abcam, Cambridge, UK), VEGF (1:2000, ab214424, Abcam), PGF (1:1000, EM1701-88, HUABIO, Woburn, MA, USA), and *β*-actin (1:5000, ab8227, Abcam). Target proteins were normalized to *β*-actin and quantified using ImageJ 1.51j8 software. 

### 2.3. Embryonic Metabolomic Analysis

#### 2.3.1. Embryo Sample Preparation

The embryos were mixed with a solution of acetonitrile and water (*v*/*v*, 1:1). After homogenizing, the samples were centrifuged at 15,000× *g* rpm for 15 min. The supernatant was filtered through a nylon membrane (0.22 μm) and transferred to auto sample vials for liquid chromatography/mass spectrometry (LC/MS) analysis. 

#### 2.3.2. LC/MS Analysis

Embryonic metabolomic analysis was performed using Agilent Technologies 6530C Q-TOF LC/MS equipped with an ACQUITY UPLC BEH C18 column (1.7 μm, 2.1 mm × 100 mm, Waters, Milford, MA, USA). The injection volume was 2 μL. The flow rate was 0.4 mL min^−1^. The mobile phase consisted of 0.1% formic acid in water (A) and acetonitrile (B). The gradient elution program was as follows: 0–3.0 min, 5–20% A, 95–80% B; 3.0–6.5 min, 20–50% A, 80–50% B; 6.5–12.5 min, 50–85% A, 50–15% B; 12.5–17.5 min, 85–100% A, 15–0% B; 17.5–23.0 min, and 100% A. The MS was set at ESI+ mode with a mass range of 50–1000 m/z. The capillary and cone voltages were 3 kV and 40 V, respectively. 

#### 2.3.3. Data Preprocessing

Metabolites were identified using online databases, including the Human Metabolome Database (HMDB), MyCompoundID, and LIPID MAPS. The raw data extracted using Agilent MassHunter were imported into MS-DIAL for preprocessing. Principal component analysis (PCA) was performed for visualizing the sample clustering. Supervised multivariate partial least-squares discriminant analysis (PLS-DA) was applied to explore the between-group variability induced by the intervention. The data were log_10_-transformed before PCA and PLS-DA. The quality of the PLS-DA models was assessed using the *R*^2^*Y* (goodness of fit) and *Q*^2^*Y* (goodness of prediction) parameters, and a permutation test was performed to further confirm the validity of the model. The variable importance on projection values (VIP) from PLS-DA, *p*-value from a Student’s *t* test, and fold change (FC) were calculated using MetaboAnalyst 5.0. Differential metabolites were identified if VIP > 1, *p* < 0.05, and FC > 2 or <0.5. We first identified differentially altered metabolites between the HC and DMC groups. If they were also differentially altered between at least one treatment group (L*n*-3, H*n*-3, or *n*-6) and the DMC group, these metabolites were considered potential candidates related to the improving effect of PUFA on placental and embryonic growth and were included in the subsequent correlation analysis.

### 2.4. Statistical Analysis

Between-group differences in protein levels, relative abundance of differentially altered metabolites, and parameters related to embryonic and placental growth were tested using a one-way ANOVA, followed by LSD post hoc analysis. Spearman correlation coefficients were calculated to evaluate the relationship between the embryonic and placental growth parameters (embryo weight, placenta weight, and weight ratio of embryo to placenta), proteins, and metabolites. All of the above analyses were conducted in SPSS (version 26.0). A *p* < 0.05 was considered to be statistically significant. Pathway and heatmap analyses were performed using MetaboAnalyst 5.0 and TBtools, respectively. PCA and PLS-DA plots were generated using SIMCA (version 14.1), and other figures were constructed using GraphPad Prism (version 8.0). The graphic abstract was partly generated using Servier Medical Art (https://smart.servier.com/), provided by Servier, licensed under a Creative Commons Attribution 3.0 Unported License. 

## 3. Results

### 3.1. Effect of Polyunsaturated Fatty Acids on Embryonic and Placental Growth

Pregnant mice provided 75 embryos in the HC group, 112 in the DMC group, 72 in the L*n*-3 group, 90 in the H*n*-3 group, and 133 in the *n*-6 group. Each embryo and placenta was analyzed as an independent unit.

The embryo weight was described in our previous study from the same batch of samples: the embryo weight in the DMC group (0.038 ± 0.018 g) was significantly lower than in the HC group (0.100 ± 0.016 g) (*p* < 0.001); the embryo weight in the H*n*-3 group (0.055 ± 0.013 g) was slightly higher than in the *n*-6 group (0.051 ± 0.025 g), although there was no significant difference (*p* > 0.05); the embryo weights in the H*n*-3 and *n*-6 groups were significantly higher than in the DMC group (*p* < 0.001); no significant difference in embryo weight was observed between the L*n*-3 (0.035 ± 0.014 g) and DMC groups (*p* > 0.05) [3]. 

**Figure 1:**
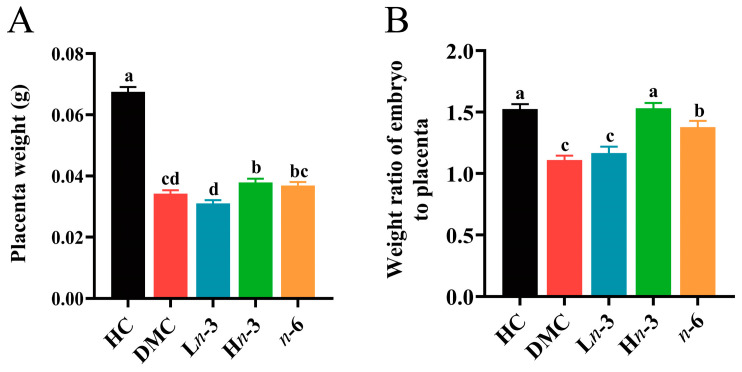
Effect of *n*-3 and *n*-6 polyunsaturated fatty acids (PUFAs) on placenta weight and weight ratio of embryo to placenta. *n* = 75, 112, 72, 90, and 133 for HC, DMC, L*n*-3, H*n*-3, and *n*-6 groups, respectively. HC, healthy mice + normal diet (AIN-93G); DMC, diabetic mice + normal diet; L*n*-3, diabetes + diet of low-dose *n*-3 PUFA; H*n*-3, diabetes + diet of high-dose *n*-3 PUFA; *n*-6, diabetes + diet of *n*-6 PUFA. Lowercase letters a–d marked on each group indicate different levels of placenta weight and weight ratio of embryo to placenta, and “a” means the highest level; significant difference observed between groups that do not share the same letter. (**A**) placenta weight in different groups; (**B**) weight ratio of embryo to placenta in different groups.

Compared with the HC group, the placenta weight and the weight ratio of embryo to placenta were significantly lower in the DMC group (*p* < 0.001). The placenta weight in the DMC group was lower than in the H*n*-3 group (*p* < 0.05) and was comparable with the L*n*-3 and *n*-6 groups (*p* > 0.05) (Figure 1A). The weight ratio of embryo to placenta in the *n*-6 group was higher than in the DMC group (*p* < 0.001) and was lower than in the H*n*-3 group (*p* < 0.05). No significant difference was observed in the weight ratio of embryo to placenta between the L*n*-3 and DMC groups (*p* > 0.05) (Figure 1B). 

### 3.2. Effects of Polyunsaturated Fatty Acids on Embryonic Metabolites 

**Figure 2:**
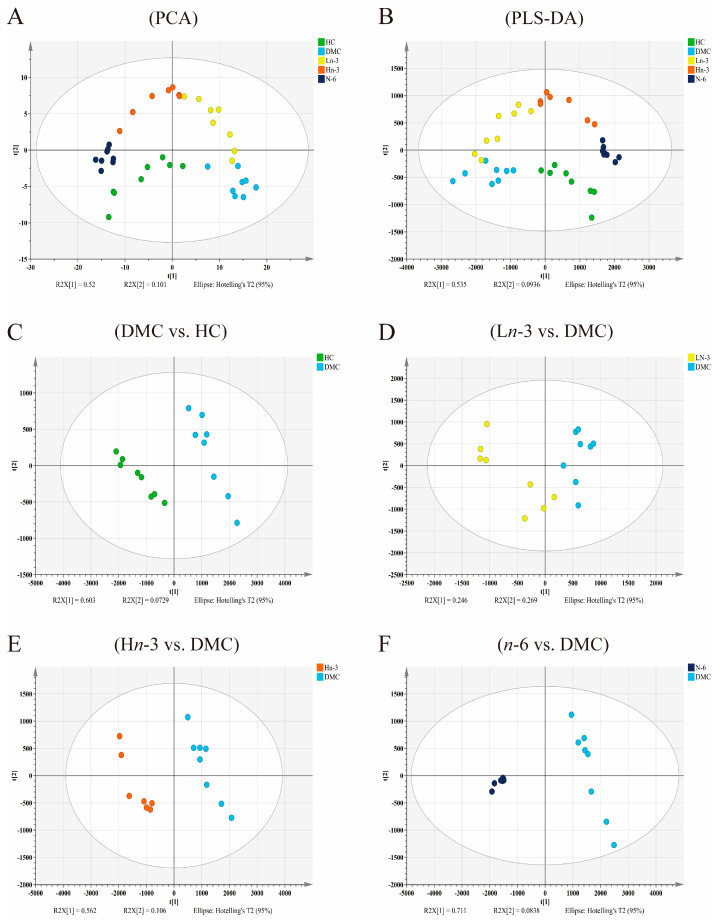
Score plots of PCA and PLS-DA of five groups (**A**,**B**) and score scatter plots of the PLS-DA model for pairwise comparison between groups (**C**–**F**). *n* = 8 for HC, DMC, L*n*-3, and *n*-6 groups, *n* = 7 for H*n*-3 group. HC, healthy mice + normal diet (AIN-93G); DMC, diabetic mice + normal diet; Ln-3, diabetes + diet of low-dose *n*-3 PUFA; Hn-3, diabetes + diet of high-dose *n*-3 PUFA; *n*-6, diabetes + diet of *n*-6 PUFA. PCA, principal component analysis; PLS-DA, partial least-squares discriminant analysis.

A clear distinction was observed between different groups in the PCA and PLS-DA models, indicating that there was a significant difference in the metabolites between groups (Figure 2). The *R*^2^*Y* and *Q*^2^*Y* values of each PLS-DA model are shown in Table 1. All *Q*^2^*Y* values from the PLS-DA models were higher than 0.6, indicating the good quality of these models. All *R*^2^*Y* and *Q*^2^*Y* values generated using the permutation test were smaller than that in the actual model (generated by the PLS-DA model), indicating that the PLS-DA model had good predictive power without overfitting (Figure S1). 

**Figure 3:**
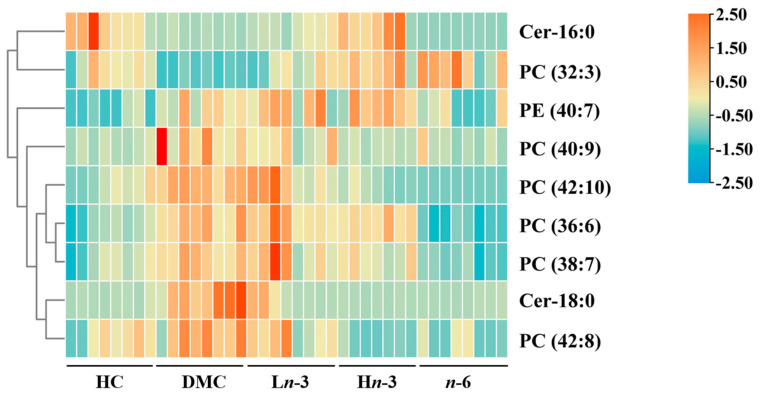
Heatmap analysis of 9 differentially altered embryonic metabolites. *n* = 8 for HC, DMC, L*n*-3, and *n*-6 groups, *n* = 7 for H*n*-3 group. The data were row-standardized to investigate the change trend of metabolites among the five groups. HC, healthy mice + normal diet (AIN-93G); DMC, diabetic mice + normal diet; L*n*-3, diabetes + diet of low-dose *n*-3 PUFA; H*n*-3, diabetes + diet of high-dose *n*-3 PUFA; *n*-6, diabetes + diet of *n*-6 PUFA.

A total of 219 differential metabolites were identified between the HC and DMC groups. Among these, the relative peak intensities of 44 metabolites were significantly different between the treatment groups and the DMC group. Of these 44 metabolites, nine were matched with databases (HMDB, MyCompoundID, and LIPID MAPS) (mass tolerance ≤ 0.05 Da), including seven glycerophospholipids and two sphingolipids. Their heatmap is shown in Figure 3. Among these nine differential metabolites, two (PC (32:3) and Cer-16:0) were significantly lower, and seven (PC (36:6), PC (38:7), PC (40:9), PC (42:8), PC (42:10), PE (40:7), and Cer-18:0) were significantly higher in the DMC group than in the HC group (*p* < 0.05) (Table 2). PC (32:3) was significantly higher in the L*n*-3, H*n*-3, and *n*-6 groups than in the DMC group (*p* < 0.05). PC (36:6), PC (38:7), and PE (40:7) were significantly lower in the *n*-6 group than in the DMC group (*p* < 0.001). PC (40:9) and Cer-18:0 were significantly lower in the L*n*-3, H*n*-3, and *n*-6 groups than in the DMC group (*p* < 0.05). PC (42:8) and PC (42:10) were significantly lower in the H*n*-3 and *n*-6 groups than in the DMC group (*p* < 0.001). Cer-16:0 was significantly higher in the H*n*-3 group than in the DMC group (*p* < 0.001). Compared with the *n*-6 group, the H*n*-3 group had higher levels of Cer-16:0, PC (36:6), PC (38:7), and PE (40:7) (*p* < 0.05).

### 3.3. Metabolic Pathway Analysis

**Figure 4:**
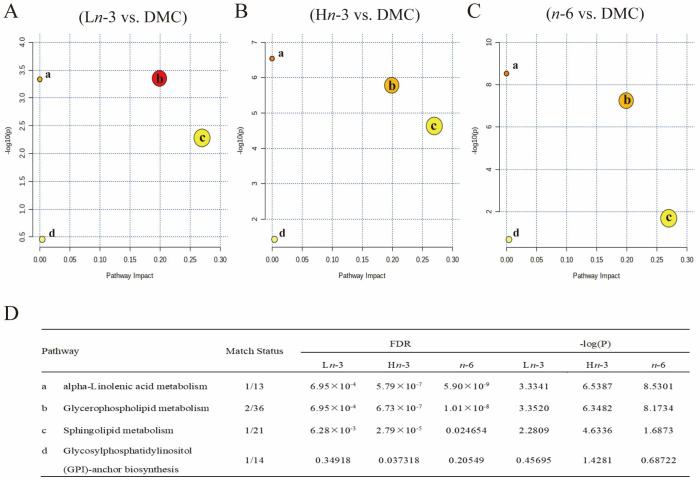
Pathway analysis of differentially altered embryonic metabolites. *n* = 8 for HC, DMC, L*n*-3, and *n*-6 groups, *n* = 7 for H*n*-3 group. HC, healthy mice + normal diet (AIN-93G); DMC, diabetic mice + normal diet; L*n*-3, diabetes + diet of low-dose *n*-3 PUFA; H*n*-3, diabetes + diet of high-dose *n*-3 PUFA; *n*-6, diabetes + diet of *n*-6 PUFA. FDR, false discovery rate. (**A**–**C**), pathway analysis for L*n*-3 vs. DMC (**A**), H*n*-3 vs. DMC (**B**), and *n*-6 vs. DMC (**C**). (**D**) FDR and -log(P) for pathways. a–d in (**A**–**C**) indicated the main affected pathways, and their names were shown in (**D**). Different colors in (**A**–**C**) indicated different levels of significance, and a deeper color indicated a greater -log(P) value.

We conducted an enrichment analysis of multiple metabolic pathways using MetaboAnalyst (Figure 4). The main metabolic pathways affected by *n*-3 and *n*-6 PUFAs were related to alpha-linolenic metabolism, glycerophospholipid metabolism, sphingolipid metabolism, and glycosylphosphatidylinositol anchor biosynthesis. 

### 3.4. Correlation Analysis of Embryonic Metabolites with Embryo Weight, Placenta Weight, and Weight Ratio of Embryo to Placenta

**Figure 5:**
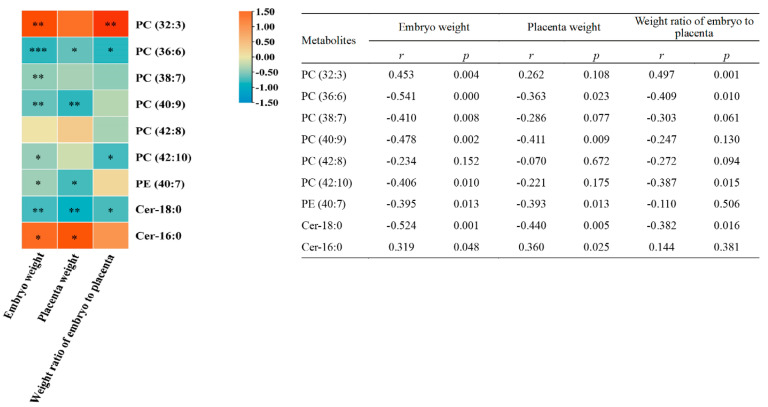
Correlation analysis of the 9 differential embryonic metabolites with embryo weight, placenta weight, and weight ratio of embryo to placenta (*n* = 39). The intensity of colors in the heatmap represents the degree of association as measured by Spearman’s correlations. * *p* < 0.05; ** *p* < 0.01; *** *p* < 0.001.

The levels of eight of the nine differential metabolites were strongly related to embryo weight: the embryo weight was positively correlated with levels of PC (32:3) and Cer-16:0 (*r* = 0.453, *p* = 0.004; *r* = 0.319, *p* = 0.048) and negatively correlated with the other six metabolites (PC (36:6), PC (38:7), PC (40:9), PC (42:10), PE (40:7), and Cer-18:0) (Figure 5). Placenta weight was positively correlated with the level of Cer-16:0 (*r* = 0.360, *p* = 0.025), and negatively correlated with the levels of PC (36:6) (*r* = −0.363, *p* = 0.023), PC (40:9) (*r* = −0.411, *p* = 0.009), PE (40:7) (*r* = −0.393, *p* = 0.013), and Cer-18:0 (*r* = −0.440, *p* = 0.005). The weight ratio of embryo to placenta was positively correlated with the level of PC (32:3) (*r* = 0.497, *p* = 0.001) and negatively correlated with the levels of PC (36:6) (*r* = −0.409, *p* = 0.010), PC (42:10) (*r* = −0.387, *p* = 0.015), and Cer-18:0 (*r* = −0.382, *p* = 0.016).

### 3.5. Effect of Polyunsaturated Fatty Acids on Placental Growth Factors

**Figure 6:**
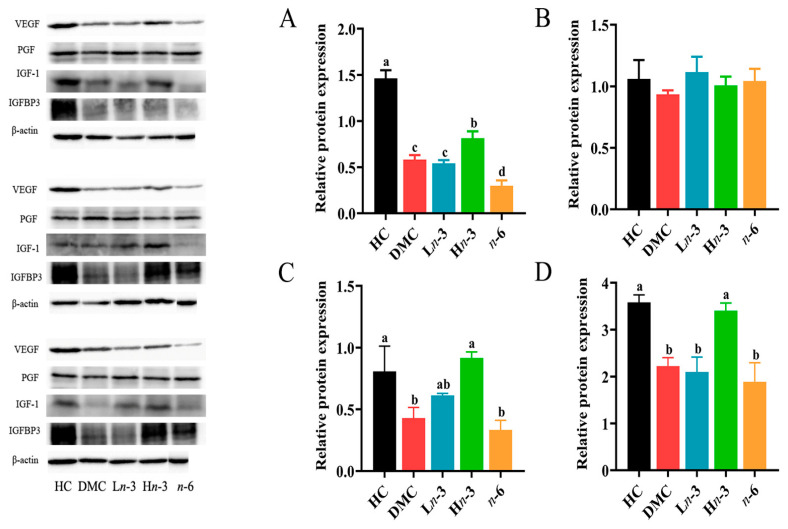
Effect of *n*-3 and *n*-6 polyunsaturated fatty acid (PUFA) supplementation during pregnancy on protein expression of placental growth factors (*n* = 3 in each group). Representative bands of Western blotting analysis were cropped from the raw images provided in the Supplementary Materials. (**A**–**D**), relative contents of VEGF (**A**), PGF (**B**), IGF-1 (**C**), and IGFBP3 (**D**). VEGF, vascular endothelial growth factor; PGF, placental growth factor; IGF-1, insulin-like growth factor-1; IGFBP3, IGF-binding protein 3. HC, healthy mice + normal diet (AIN-93G); DMC, diabetic mice + normal diet; L*n*-3, diabetes + diet of low-dose *n*-3 PUFA; H*n*-3, diabetes + diet of high-dose *n*-3 PUFA; *n*-6, diabetes + diet of *n*-6 PUFA. Lowercase letters a–d marked on each group indicate different levels of proteins, and “a” means the highest level; significant difference observed between groups that do not share the same letter.

As shown in Figure 6, the levels of VEGF, IGF-1, and IGFBP3 proteins in the DMC group were lower than in the HC group (*p* < 0.05) and were comparable with the L*n*-3 group (*p* > 0.05). The levels of VEGF, IGF-1, and IGFBP3 in the H*n*-3 group were higher than in the DMC group (*p* < 0.05). Compared with the DMC group, the *n*-6 group had a significantly lower level of VEGF (*p* < 0.05) and comparable levels of IGF-1 and IGFBP3 (*p* > 0.05). No significant group difference was observed for PGF protein levels (*p* > 0.05).

### 3.6. Correlation Analysis of Placental Growth Factors with Embryo Weight, Placenta Weight, and Weight Ratio of Embryo to Placenta

The embryo and placenta weights were positively correlated with the protein levels of VEGF (*r* = 0.579, *p* = 0.024; *r* = 0.686, *p* = 0.005) and IGFBP3 (*r* = 0.646, *p* = 0.009; *r* = 0.800, *p* < 0.001) (Table 3). 

### 3.7. Correlation Analysis of Embryo Differential Metabolites with Placental Growth Factors

**Figure 7:**
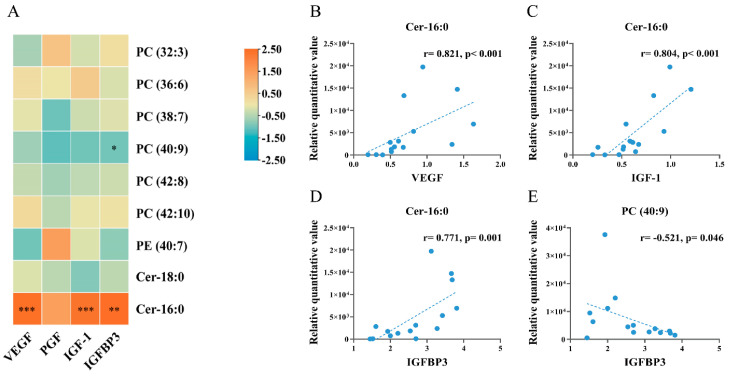
Correlation analysis of differentially altered embryonic metabolites with protein levels of placental growth factors (*n* = 15). (**A**) heat map for correlation analysis; (**B**,**C**), scatter plots showing the correlations between Cer-16:0 and VEGF (**B**), between Cer-16:0 and IGF-1 (**C**), between Cer-16:0 and IGFBP3 (**D**), between PC (40:9) and IGFBP3 (**E**). In scatter plots, each dot indicated a sample, and the dashed line indicated the linear fit line. Spearman correlation coefficients were calculated for the relationships between metabolites and placental protein levels. * *p* < 0.05; ** *p* < 0.01; *** *p* < 0.001.

Among the nine differential metabolites, two were strongly associated with placental growth factor levels: the level of Cer-16:0 was positively correlated with the protein levels of VEGF (*r* = 0.821, *p* < 0.001), IGF-1 (*r* = 0.804, *p* < 0.001), and IGFBP3 (*r* = 0.771, *p* = 0.001); the level of PC (40:9) was negatively correlated with the protein level of IGFBP3 (*r* = −0.521, *p* = 0.046) (Figure 7).

## 4. Discussion

*n*-3, but not *n*-6, PUFA may promote placental and embryonic growth through placental growth factors. In the present study, *n*-3 PUFA improved the protein expression of placental VEGF, IGF-1, and IGFBP3, and VEGF and IGFBP3 showed positive correlations with embryo and placenta weight. These results suggest that *n*-3 PUFA may promote placental and embryonic growth by increasing the expression of placental growth factors. Previous studies have also found that *n*-3 PUFA supplementation increased the mRNA and protein levels of VEGF in first-trimester trophoblast cells and skeletal muscle IGF-1 mRNA levels in pigs, and the plasma concentration of IGF-1 in heifers [25,26,29]. In the present study, the *n*-6 group had a significantly lower VEGF and comparable IGF-1 and IGFBP3 levels compared with the DMC group, indicating that *n*-6 PUFA did not improve placental growth and development. As the interface between mother and fetus, the placenta mediates the exchange of nutrients, oxygen, and waste products to maintain normal fetal growth and development [30]. VEGF is involved in the regulation of placental vasculogenesis and angiogenesis processes [31]. Previous studies have observed that vascular growth increased placental–fetal blood flow in ovine placenta and IGF-1 stimulated glucose and amino acid uptake in human trophoblasts [32,33]. Therefore, the increase in placental growth factors by *n*-3 PUFA in the present study may benefit the maternal–fetal transport system. In addition, as reported in our previous study, there was no significant difference in blood glucose level at both E8.5d and E12.5d among the four diabetic groups [3], indicating that the beneficial effect of *n*-3 PUFA on placental and embryonic growth was not attributed to its hypoglycemic effect.

*n*-3, but not *n*-6, PUFA may promote embryonic growth by regulating embryonic Cer-16:0. We observed that a high dose of *n*-3 PUFA, but not *n*-6 PUFA, supplementation promoted the formation of embryonic Cer-16:0. Previous studies also found that *n*-3 and *n*-6 PUFAs might have different effects on ceramides in adipose tissue or serum. Fish oil supplementation significantly increased the Cer-24:1 level in the adipose tissue of high-fat-diet-fed rats [34]. A randomized controlled trial found that the *n*-6 PUFA (sunflower oil) group had lower serum levels of Cer-18:0, Cer-20:0, and Cer-24:1 in overweight patients compared with the saturated fatty acids (SFAs) (palm oil) group [35]. Although the two studies examined ceramide levels in different biological samples (rat adipose tissue and human serum), the results revealed different modulatory effects of *n*-3 and *n*-6 PUFAs on the level of ceramides. The different effect of *n*-3 and *n*-6 PUFAs on Cer-16:0 observed in the present study has not been reported by any previous study. However, abnormal ceramide levels in the placenta may cause the development of FGR [15,36]. A case–control study found that Cer-18:0, Cer-20:0, and Cer-24:0 levels increased in the placentas of patients with pre-eclampsia, a disorder that is commonly associated with growth restriction [36]. In the present study, *n*-3, but not *n*-6, PUFA increased Cer-16:0, and Cer-16:0 was positively correlated with embryo and placenta weight, indicating that *n*-3, but not *n*-6, PUFA may promote embryonic growth by regulating embryonic Cer-16:0. In addition, we found a positive correlation between embryonic Cer-16:0 and placental VEGF and IGFBP3 levels, suggesting that *n*-3 PUFA may increase the level of Cer-16:0 by promoting the expression of VEGF and IGFBP3.

We observed that *n*-6 PUFA supplementation ameliorated the embryonic growth retardation induced by maternal diabetes compared with the DMC group. Compared with soybean oil in the normal diet (DMC group), corn oil in the *n*-6 PUFA diet (*n*-6 group) had a much lower C18:3*n*-3 content and slightly higher C18:2*n*-6 content in the present study [3]. In the biosynthesis of long-chain polyunsaturated fatty acids (LC-PUFA), C18:3*n*-3 and C18:2*n*-6 compete for Δ-6 desaturase, catalyzing the biosynthesis of *n*-3 LC-PUFA (such as C22:6*n*-3) from C18:3*n*-3 and *n*-6 LC-PUFA (such as C20:4*n*-6) from C18:2*n*-6 [37]. A low concentration of C18:3*n*-3 would increase the biosynthesis of *n*-6 LC-PUFA, leading to an increase in C20:4*n*-6 content. Indeed, we observed that C20:4*n*-6 was higher in the *n*-6 group in the maternal serum and embryos compared with the diabetic control group, and this was also reported in our previous study with the same batch of samples [3]. Previous studies have reported a positive association between cord blood C20:4*n*-6 and gestational duration and infant birth weight [38], and C20:4*n*-6 is known to reverse the growth failure associated with essential fatty acid deficiency in rats [39]. In addition, a previous study also observed a positive correlation between birth weight and the neonatal plasma content of C20:4*n*-6 and total *n*-6 PUFA in preterm infants, suggesting that C20:4*n*-6 is beneficial for embryonic growth [40].

The beneficial effect of *n*-3 and *n*-6 PUFAs on embryonic growth may be related to embryonic PC and ceramide. In the present study, we observed that a high dose of *n*-3 PUFA and *n*-6 PUFA supplementation increased the level of PC (32:3), and decreased the levels of PC (40:9), PC (42:10), and Cer-18:0. A previous study found that fish oil supplementation increased serum PC (35:5) and PC (36:5) levels and decreased PC (36:6) and PC (38:1) levels in patients with nonalcoholic fatty liver disease (NAFLD) [41]. Moreover, the C20:4n-6 enriched diet significantly decreased the hepatic levels of PC containing C18:2n-6 in mice compared with the control diet [42]. Although previous studies have evaluated the effects of *n*-3 and *n*-6 PUFA on PC in the serum and liver tissue [41,42], the effects on embryonic PC have not been reported. In addition, *n*-3 PUFA supplementation was observed to reduce total hepatic ceramide levels and Cer-18:0 levels in a pre-diabetic rat model, which is partially consistent with our results regarding Cer-18:0 [43]. The effects of *n*-3 and *n*-6 PUFAs on embryonic Cer-18:0 have not been reported previously. In the present study, embryo weight and weight ratio of embryo to placenta were positively correlated with embryonic PC (32:3) and negatively correlated with PC (42:10) and Cer-18:0. These results indicate that *n*-3 and *n*-6 PUFAs may promote embryonic growth by regulating embryonic PC and ceramide.

*n*-3 and *n*-6 PUFAs had different regulatory effects on embryonic PC (36:6), PC (38:7), and PE (40:7). In the present study, *n*-6, but not *n*-3, PUFA supplementation decreased embryonic PC (36:6), PC (38:7), and PE (40:7) levels. Previous studies have also found that *n*-3 and *n*-6 PUFAs might have different effects on PC and PE. Wild-type mice fed a diet rich in *n*-6 PUFA had higher plasma PC (40:4), PC (38:5), and PE (40:5) levels and lower PC (38:7) and PC (42:10) levels than fat-1 transgenic mice fed the same diet, which can convert *n*-6 into *n*-3 PUFA [44]. Moreover, a randomized controlled trial on healthy subjects observed that PM_2.5_ concentration was positively associated with serum PC (21:0), PC (36:4), and PC (42:3) levels in the *n*-3 PUFA group (fish oil), while the association became negative in the *n*-6 PUFA group (sunflower seed oil) [45]. Several studies have reported that FGR is characterized by abnormal PC levels in the cord blood [13,46]. In the present study, we found that embryo weight was negatively correlated with PC (36:6), PC (38:7), and PE (40:7), and *n*-6 PUFA decreased PC (36:6), PC (38:7), and PE (40:7) levels. These results indicate that *n*-6 PUFA may promote embryonic growth by regulating embryonic PC (36:6), PC (38:7), and PE (40:7) levels.

In the present study, we observed that the weight ratio of embryo to placenta in the Hn-3 group was significantly higher than in the *n*-6 group, suggesting that the placenta in the Hn-3 group was more efficient in transporting oxygen and nutrients to the fetus. In addition, our previous study of the same batch of samples found that the embryo weight in E12.5d embryos in the high-dose *n*-3 PUFA group was slightly higher than in the *n*-6 PUFA group, although there was no significant difference [3]. Our previous study found that the embryo weight in E18.5d embryos in the *n*-6 PUFA group was significantly lower than in the *n*-3 PUFA group and was comparable with the sodium valproate control group in a mouse model of sodium-valproate-induced neural tube defects [47]. The short duration of the intervention may explain why the embryo weight in the Hn-3 group in the present study was slightly, but not significantly, higher than that in the *n*-6 group.

Maternal diabetes is associated with an increased risk of macrosomia and large birthweight for gestational age [2,48]. However, in polytocous laboratory animals such as rodents, maternal diabetes has been found to be associated with FGR [49,50,51]. In human twin pregnancies, good maternal glycemic control is associated with a higher risk of babies being small for their gestational age in subjects with diabetes than in non-diabetic controls [52]. These results indicate that pregnancies with multiple embryos may exert different influences on fetal growth, and possible reasons may include a larger placental mass, higher levels of placental hormones, earlier gestational age at birth, and slower fetal growth in polytocous pregnancy than in singleton pregnancy [52]. Therefore, increasing embryonic growth is meaningful for polytocous diabetic pregnancy and should not be used for singleton diabetic pregnancy.

The present study had some limitations. Firstly, this was an animal study, and the extrapolation of the results to humans requires caution. Secondly, many differential metabolites in this study could not be identified through online databases. Therefore, it is necessary to identify these metabolites and explore their relationship with placental and embryonic growth in future studies.

## 5. Conclusions

In conclusion, *n*-3 PUFA and *n*-6 PUFA improved placental and embryonic growth through different mechanisms.

## Figures and Tables

**Table 1:** *R*^2^*Y* and *Q*^2^*Y* values of the PLS-DA model for embryonic metabolites.

	*R* ^2^ *Y*	*Q* ^2^ *Y*
DMC vs. HC	0.92	0.88
L*n*-3 vs. DMC	0.91	0.68
H*n*-3 vs. DMC	0.96	0.95
*n*-6 vs. DMC	0.98	0.98

HC, healthy mice + normal diet (AIN-93G); DMC, diabetic mice + normal diet; L*n*-3, diabetes + diet of low-dose *n*-3 PUFA; H*n*-3, diabetes + diet of high-dose *n*-3 PUFA; *n*-6, diabetes + diet of *n*-6 PUFA.

**Table 2:** Differential metabolites in embryos across groups.

Lipid Class	Compound	m/z	Formula	Changing Trend				
				DMC vs. HC	L*n*-3 vs. DMC	H*n*-3 vs. DMC	*n*-6 vs. DMC	H*n*-3 vs. *n*-6
Glycerophospholipids								
Phosphatidylcholine								
	PC (32:3)	728.5178	C_40_H_74_NO_8_P	↓ **	↑ *	↑ ***	↑ ***	-
	PC (36:6)	778.5412	C_44_H_76_NO_8_P	↑ ***	-	-	↓ ***	↑ ###
	PC (38:7)	804.5558	C_46_H_78_NO_8_P	↑ **	-	-	↓ ***	↑ #
	PC (40:9)	828.5541	C_48_H_78_NO_8_P	↑ **	↓ *	↓ **	↓ **	-
	PC (42:8)	858.5974	C_50_H_84_NO_8_P	↑ **	-	↓ ***	↓ ***	-
	PC (42:10)	854.5692	C_50_H_80_NO_8_P	↑ ***	-	↓ ***	↓ ***	-
								
Phosphatidylethanolamine	PE (40:7)	790.554	C_45_H_76_NO_8_P	↑ **	-	-	↓ *	↑ #
								
Sphingolipids	Cer-18:0	566.5499	C_36_H_71_NO_3_	↑ ***	↓ ***	↓ ***	↓ ***	-
	Cer-16:0	538.5176	C_34_H_67_NO_3_	↓ ***	-	↑ ***	-	↑ ###

*n* = 8 for HC, DMC, L*n*-3, and *n*-6 groups, *n* = 7 for H*n*-3 group. The “↑” and “↓” arrows represent a significant increasing or decreasing trend of embryonic metabolites. “-” means no significant change. Significant differences were determined by a one-way ANOVA followed by an LSD analysis. HC, healthy mice + normal diet (AIN-93G); DMC, diabetic mice + normal diet; L*n*-3, diabetes + diet of low-dose *n*-3 PUFA; H*n*-3, diabetes + diet of high-dose *n*-3 PUFA; *n*-6, diabetes + diet of *n*-6 PUFA. Green and red arrows indicate lower and higher, respectively. * *p* < 0.05, ** *p* < 0.01, *** *p* < 0.001 vs. DMC; # *p* < 0.05, ### *p* < 0.001, H*n*-3 vs. *n*-6.

**Table 3:** Correlation analysis of placental growth factors with embryo weight, placenta weight, and weight ratio of embryo to placenta (*n* = 15).

Parameters	Embryo Weight		Placenta Weight		Weight Ratio of Embryo to Placenta	
	*r*	*p*	*r*	*p*	*r*	*p*
VEGF (relative protein level)	0.579	0.024	0.686	0.005	0.039	0.889
PGF (relative protein level)	0.036	0.899	−0.207	0.459	0.018	0.950
IGF-1 (relative protein level)	0.236	0.398	0.346	0.206	−0.143	0.612
IGFBP3 (relative protein level)	0.646	0.009	0.800	<0.001	0.089	0.752

*r*- and *p*-values were calculated using Spearman’s rank correlation. VEGF, vascular endothelial growth factor; PGF, placental growth factor; IGF-1, insulin-like growth factor-1; IGFBP3, IGF-binding protein 3.

## Data Availability

Data are contained within the article.

## Supplementary Materials

The following supporting information can be downloaded at: https://www.mdpi.com/article/10.3390/nu16081182/s1, Figure S1. Permutation tests for pairwise PLS-DA between between DMC and HC (A), L*n*-3 (B), H*n*-3 (C) and *n*-6 (D). All *R2Y* and *Q2Y* values were smaller than that in the actual model (generated by PLS-DA model). These results showed that the PLS-DA model had good predictive power without over-fitting. HC, healthy mice + normal diet (AIN-93G); DMC, diabetic mice + normal diet; L*n*-3, diabetes + diet of low-dose *n*-3 PUFA; H*n*-3, diabetes + diet of high-dose *n*-3 PUFA; *n*-6, diabetes + diet of *n*-6 PUFA.
