# Supplementary material for: Differential Effects of n-3 and n-6 Polyunsaturated Fatty Acids on Placental and Embryonic Growth and Development in Diabetic Pregnant Mice
Source: Nutrients. 2024 Apr 16;16(8):1182. doi: 10.3390/nu16081182 (PMC11054179; doi:10.3390/nu16081182)
Supplement: Supplementary file 1 [file nutrients-16-01182-s001.zip › nutrients-2888013-supplementary.pdf]

Supplementary Figure

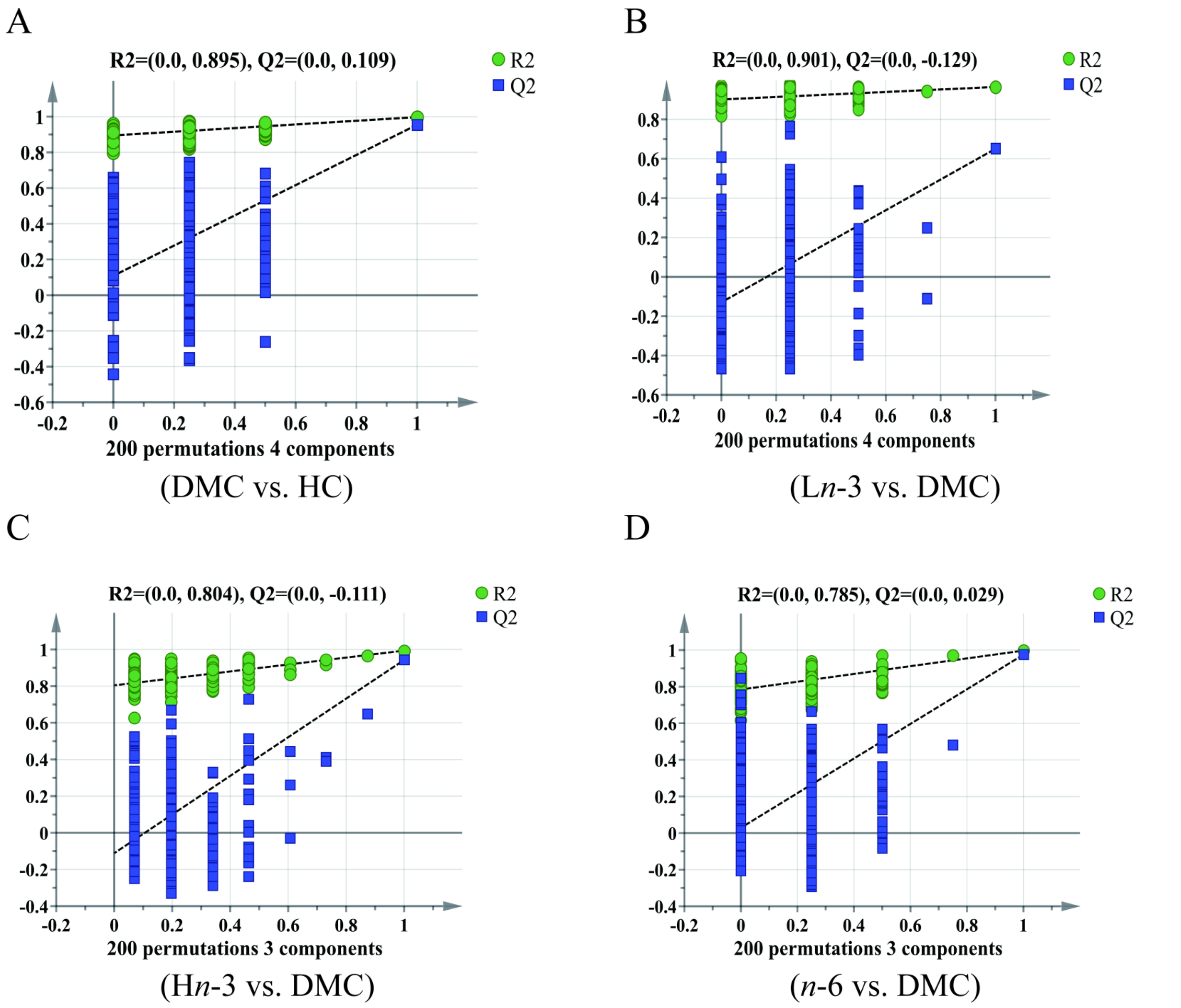

Figure S1. Permutation tests for pairwise PLS-DA between DMC and HC (**A**), L*n*-3 (**B**), H*n*-3 (**C**) and *n*-6 (**D**). All R^2Y and Q^2Y values were smaller than that in the actual model (generated by PLS-DA model). These results showed that the PLS-DA model had good predictive power without over-fitting. HC, healthy mice + normal diet (AIN-93G); DMC, diabetic mice + normal diet; L*n*-3, diabetes + diet of low-dose *n*-3 PUFA; H*n*-3, diabetes + diet of high-dose *n*-3 PUFA; *n*-6, diabetes + diet of *n*-6 PUFA.

Raw bands for western blotting analysis

Group：HC, healthy mice + normal diet (AIN-93G); DMC, diabetic mice + normal diet; L*n*-3, diabetes + diet of low-dose *n*-3 PUFA; H*n*-3, diabetes + diet of high-dose *n*-3 PUFA; *n*-6, diabetes + diet of *n*-6 PUFA

VEGF: 40 KDa; PGF: 25 KDa; IGF-1: 13 KDa; IGFBP3: 29 KDa and 46 KDa

VEGF

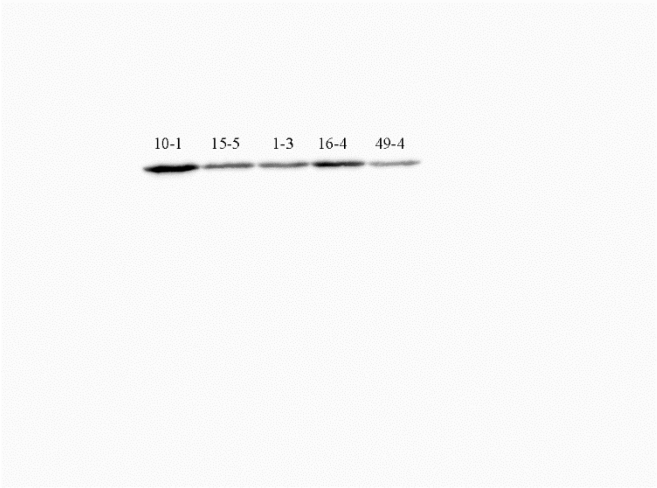

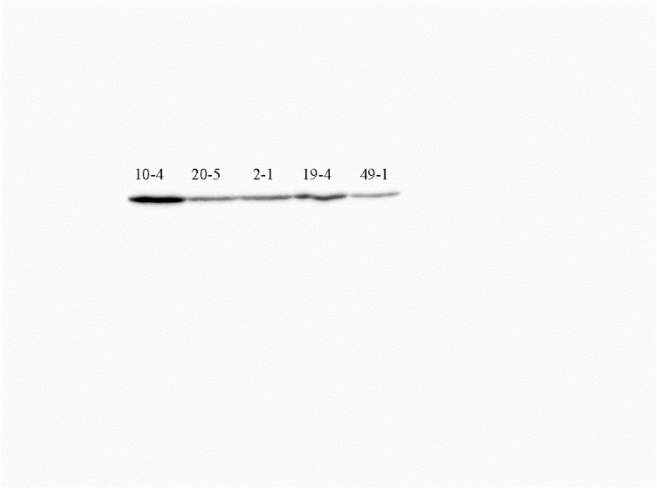

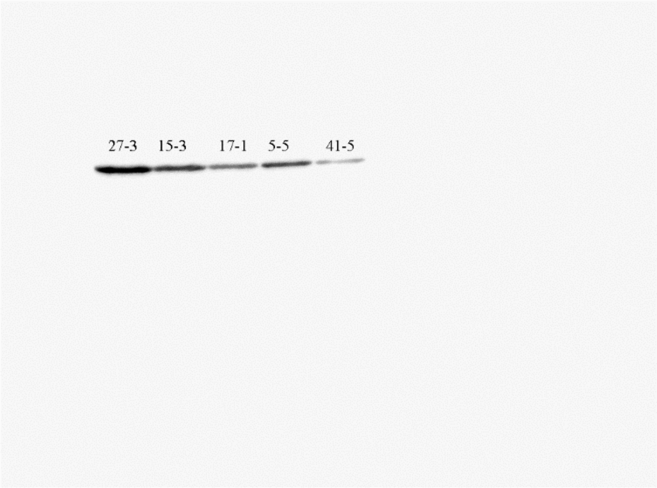

PGF

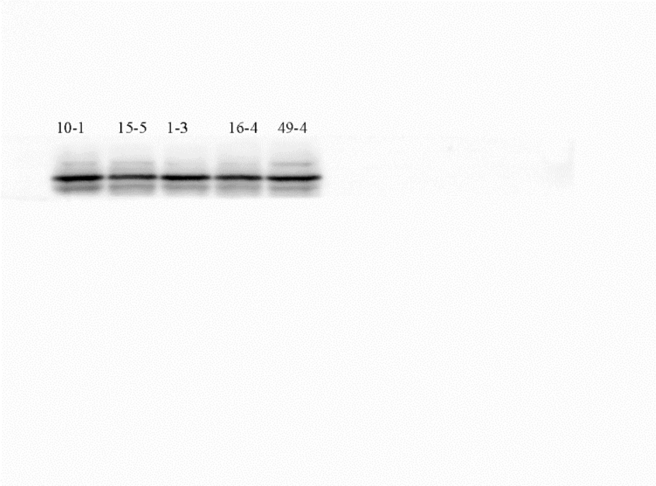

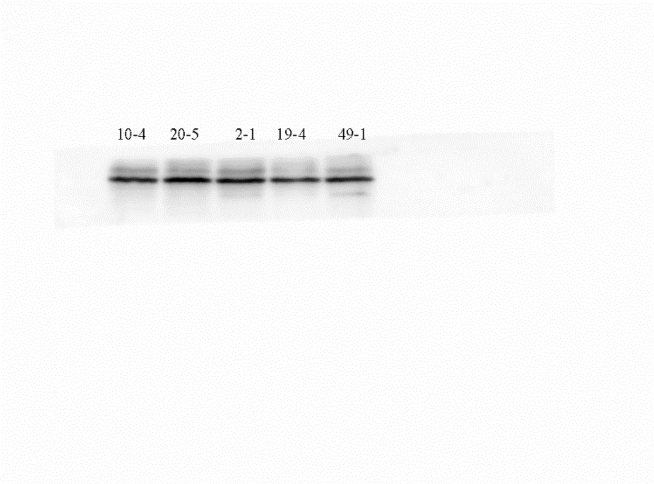

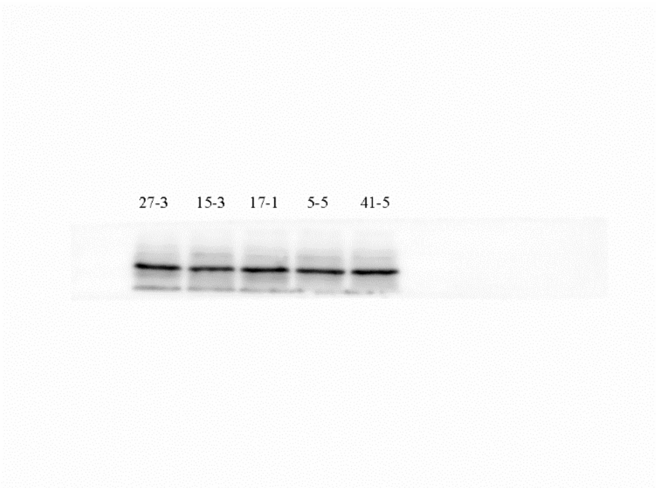

IGF-1

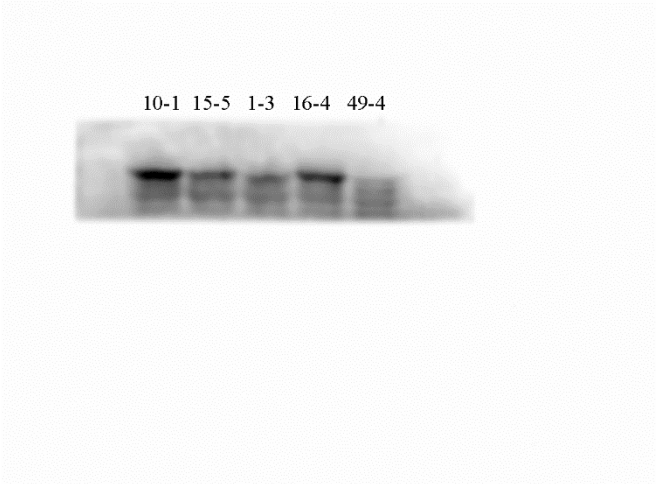

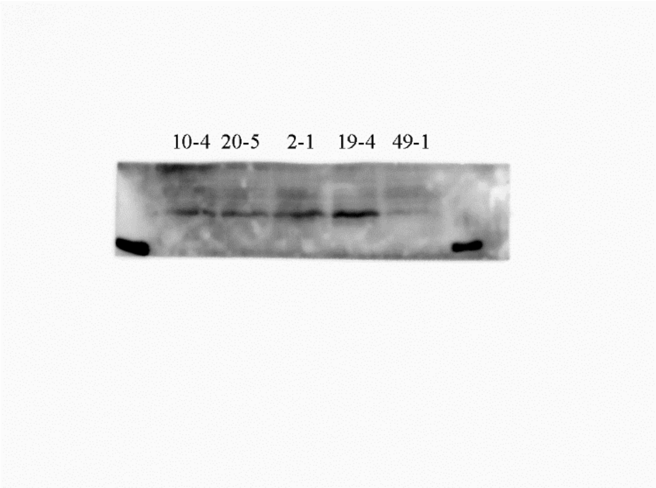

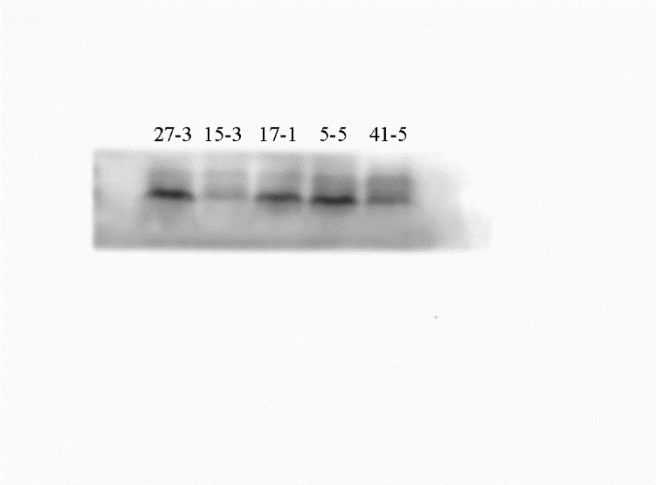

IGFBP3

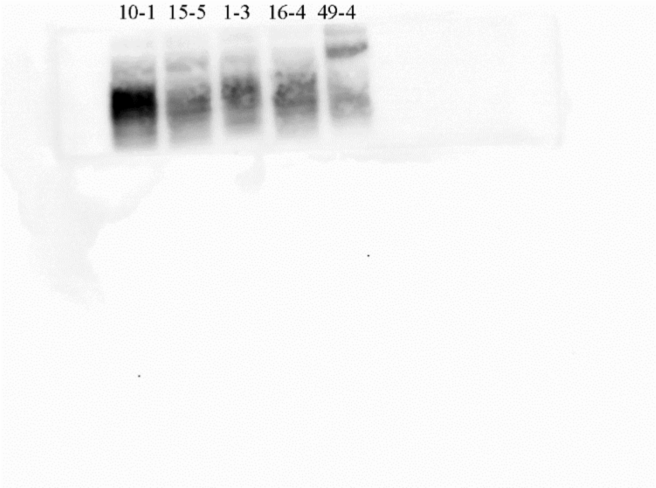

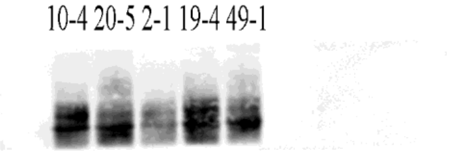

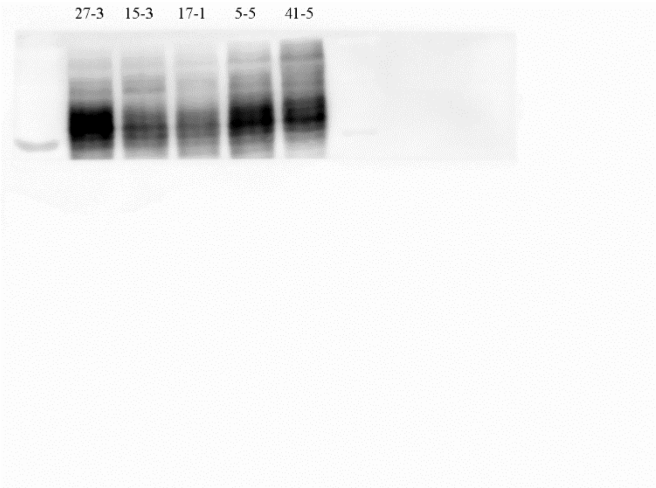

β-actin

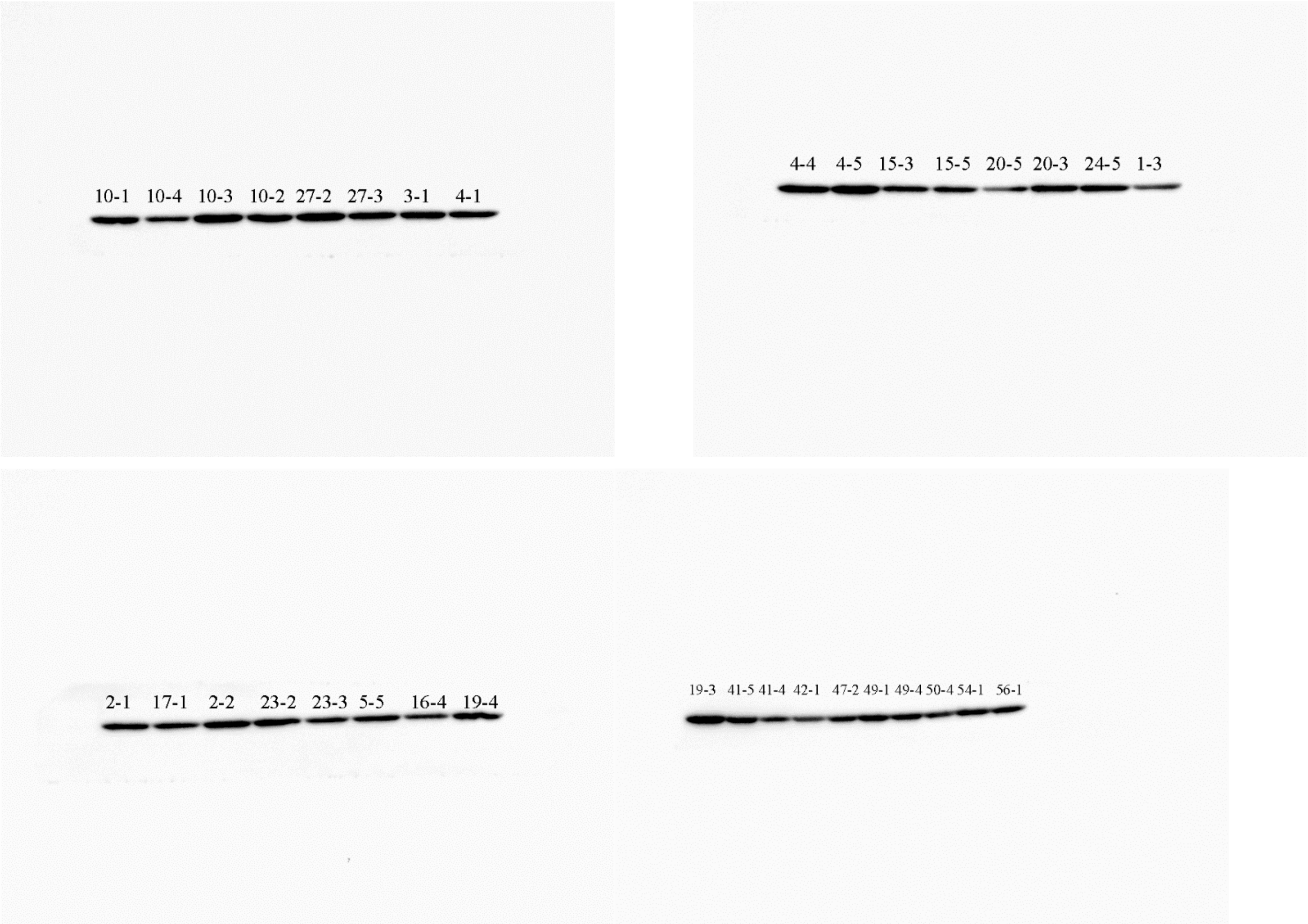
10-1 10-4 10-3 10-2 27-2 27-3 3-1 4-1
4-4 4-5 15-3 15-5 20-5 20-3 24-5 1-3
2-1 17-1 2-2 23-2 23-3 5-5 16-4 19-4
19-3 41-5 41-4 42-1 47-2 49-1 49-4 50-4 54-1 56-1
